# Survival Outcomes and Factors Associated with Revision Surgery for Metastatic Disease of the Spine

**DOI:** 10.1155/2018/6140381

**Published:** 2018-06-25

**Authors:** Vignesh K. Alamanda, Myra M. Robinson, Jeffrey S. Kneisl, Leo R. Spector, Joshua C. Patt

**Affiliations:** ^1^Department of Orthopaedic Surgery, Carolinas Medical Center, Atrium Health, Charlotte, NC, USA; ^2^Department of Cancer Biostatistics, Levine Cancer Institute, Atrium Health, Charlotte, NC, USA; ^3^Department of Orthopaedic Surgery, Levine Cancer Institute, Atrium Health, Charlotte, NC, USA; ^4^OrthoCarolina's Spine Center, Charlotte, NC, USA

## Abstract

**Study Design:**

Retrospective review of a prospective database.

**Objective:**

Certain subset of patients undergoing surgical treatment for spinal metastasis will require a revision surgery in their disease course; however, factors predictive of revision surgery and survival outcomes are largely unknown. The goal of this study is to report on survival outcomes as well as factors predictive of revision surgery in this unique patient population.

**Methods:**

A total of 55 patients who met the inclusion criteria were included from January 2010 to December 2015. Twelve (22%) of these patients underwent a revision surgery. Patient and tumor characteristics were summarized and survival outcomes were evaluated using Kaplan-Meier methods and Cox proportional hazards regression.

**Results:**

Both the revision and the nonrevision groups were similarly matched with respect to spine disease burden, neurological status at time of initial presentation, primary malignancy types, and the use of adjuvant treatment modalities. Tumor progression (66.7%) was the most common reason for necessitating a revision followed by nonunion (16.7%), wound dehiscence (8.3%), and construct failure (8.3%). Following multivariate model selection procedures, smokers were found to have 3.5 times increased odds of undergoing revision compared to nonsmokers (*p* = 0.05). Analysis of survival curves showed that the median survival in the revision group was 3.0 years (95% CI: 1.5, 4.1), while the median survival in the nonrevision group was 1.5 years (95% CI: 1.1, 2.3; log-rank test, *p* = 0.105).

**Conclusion:**

Despite aggressive treatment, tumor progression is the most common reason for revision surgery. Smoking is an independent risk factor for revision. Revision surgery should be considered in patients when indicated as it does not appear to detrimentally affect survival.

## 1. Introduction

The incidence of spinal metastasis continues to increase [1]: more than 18,000 new cases are reported every year in North America alone [2]. Treatment strategies and modalities continue to improve and have resulted in tremendous improvement in overall survival rates for these patients [3–5]. As overall survival rates continue to increase, the rate of skeletal and in particular spinal metastases can be expected to increase [6]. Additionally, indications for surgical intervention are also continuing to expand [7].

Tools for estimating prognosis have been developed to aid treating physicians with management options including surgical intervention [8, 9]. When indicated, surgical treatment is not without its drawbacks. The need for revision surgery exists in this vulnerable patient population and reasons cited include surgical site infections and wound dehiscence, failure of instrumentation, and local recurrence [9]. However, not much has been established regarding the survival outcomes of those who undergo revision surgery and the factors that predispose certain patients to undergo revision surgery.

Thus, in this study, we primarily sought to explore and compare the survival outcomes of patients undergoing revision surgery as compared to patients who only underwent a primary procedure. Additionally, our secondary goal was to assess and identify reasons for revision and risk factors that could predispose such patients to needing a revision.

## 2. Methods

After obtaining Institutional Review Board approval, a retrospective cohort study of a prospective database at a major cancer center was conducted to compare patient, tumor, and survival characteristics between those patients requiring a revision surgery and those who only underwent a primary surgical procedure.

Patients undergoing surgical treatment at our institution by the senior author between January 2010 and December 2015 were identified based on a retrospective review of a prospective database and were considered for the study. Exclusion criteria were age below 18 years and nonsurgical treatment for their metastatic disease to their spine. Additionally, primary tumors of the spine undergoing surgical treatment were excluded.

A total of 85 patients with a minimum of 6-month follow-up were identified and, after applying the exclusion criteria, 55 met the inclusion criteria. Patient demographics and tumor characteristics collected included age, gender, race, smoking status, primary malignancy type, number of vertebral levels affected, and location of spinal metastases. Additionally, treatment information, including use of radiation therapy and/or chemotherapy, use of preoperative embolization, and use of allograft, was abstracted from the medical record. The presence or absence of pain, the American Spinal Injury Association (ASIA) score at time of presentation, and reason for revision were also obtained. Overall survival (OS) was measured from the date of diagnosis of spinal metastasis to date of death from any cause. Surviving patients were censored at the date of last follow-up.

Frequencies and proportions of patient and tumor characteristics were reported and compared across groups using *t*-tests or Wilcoxon rank-sum tests for continuous variables and Fisher's exact tests for categorical data. Kaplan-Meier techniques were used to estimate OS distributions and a log-rank test compared the survival distributions between those with and without subsequent revision surgery. Cox proportional hazards regression was used to determine a hazard ratio (HR) and 95% confidence interval (CI) to estimate the magnitude of the impact of revision surgery on OS. Univariate and multivariate logistic regressions assessed the impact of patient and disease characteristics on need for revision surgery and were used to determine odds ratios (OR) to estimate the magnitude of the impact of those factors on need for revision surgery. Multivariate logistic models were determined for the outcome of revision surgery (yes/no) using backward elimination and forward selection modeling procedures (significance levels of *p* = 0.10). Individual prognostic factors were identified through univariate logistic models for all potential covariates (age, race, gender, smoking status, primary malignancy type, preoperative ambulatory status, radiotherapy, chemotherapy, and presence of extraspinal metastases).

Statistical software SAS (version 9.4) was used for all data analyses. Unless otherwise noted, a 2-sided *α* = 0.05 significance level was used.

## 3. Results

### 3.1.  Table 1: Patient and Tumor Characteristics

Of the total of 85 patients that were operated on by the senior surgeon in the group from January 2010 to December 2015, 55 patients met inclusion criteria. Of these 55 patients, 12 patients (21.8%) had undergone subsequent revision surgery.

Gender and race were not statistically different between the two groups. Approximately 60% of the study population was male and approximately 72% was Caucasian. In analyzing smoking between the two groups, 75% of the patients in the revision group were smokers compared to only 42% in the nonrevision group (*p* = 0.055). Renal cell carcinoma was the prominent cancer subtype in both groups (41.7% and 23.3% in the revision and no revision groups, resp.).

Both groups had similar proportions of metastasis to the various spinal segments. In analyzing pain, no significant difference exists between the two groups: 100% of the revision surgery group had pain at time of initial presentation following diagnosis of spinal metastasis compared to 91% in the nonrevision surgery group. None of the patients who underwent a revision surgery had spinal metastasis present at time of initial cancer diagnosis compared to 28% of the patients in the nonrevision group (*p* = 0.05).

There were no differences between the two groups in terms of ASIA motor score at time of initial presentation, the use of allograft at the time of surgery, the use of chemotherapy or radiation therapy, and the use of preoperative embolization at the time surgery. The median number of vertebral levels involved in both groups was 1.0.

### 3.2.  Table 2: Reasons for Revision

The primary reason for revision surgery was tumor progression. Approximately 67%,* n* = 8, of those needing a revision had tumor progression necessitating a need for a repeat surgery. The remaining 33%,* n* = 4, of revisions were for other reasons, including nonunion, failure of construct, and/or wound dehiscence.

### 3.3. Figure 1: Survival Curve (Revision versus No Revision)

**Figure 1:**
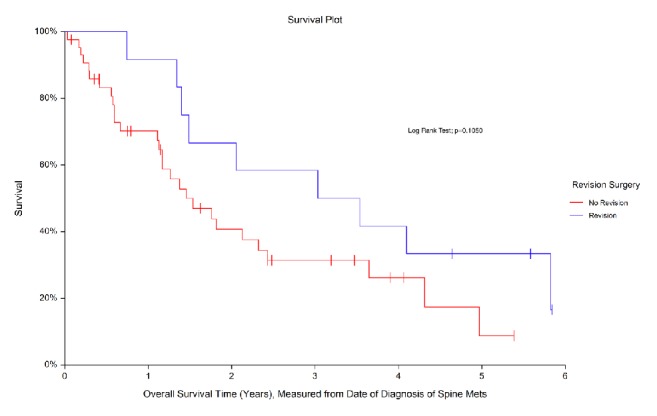


Figure 1 shows the estimated survival distributions for the two groups (revision versus no revision). The median overall survival for the group undergoing revision surgery was 3.0 years, while the median overall survival for the group that did not undergo a revision surgery was 1.5 years. Comparison of the survival curves between the two groups approached statistical significance (*p* = 0.105).

### 3.4.  Table 3: Univariate Regression Analysis for Undergoing Revision

Of the individual prognostic factors evaluated, smoking status and use of adjuvant chemotherapy individually approached statistical significant association with probability of needing a revision. Smoking was found to be individually associated with the need for a revision, as smoking increased the odds of needing a revision by 4.17 times compared to a nonsmoker (*p* = 0.052). Additionally, the odds of needing a revision in those who received adjuvant chemotherapy were 0.15 times the odds of needing a revision in those who received no adjuvant chemotherapy (*p* = 0.051).

### 3.5.  Table 4: Multivariate Regression Analysis for Undergoing Revision

When smoking status and the use of chemotherapy were modeled together in a multivariate logistic regression model, chemotherapy drops from the model and use of smoking status becomes slightly attenuated (OR: 3.52, *p* = 0.096).

In reviewing the ambulatory status of these patients, it was found that all twelve of the patients in our cohort were ambulatory either independently or with assistance prior to their operative intervention and immediately postoperatively. However, at their last clinic visit, it was found that only 5 of the 12 or 41.7% maintained their ability to ambulate.

## 4. Discussion

Surgical treatment of metastatic spine disease has been shown to improve pain, obtain control of disease, and improve quality of life [10–12]. Indications for surgical intervention for these patients continue to expand [7]. Additionally, superior adjuvant treatment options of treating metastatic disease have resulted in improved overall long-term survival of these patients [13].

Despite improvements in surgical control of spinal metastasis including obtaining circumferential spinal cord decompression and improved stabilization techniques [14], a certain proportion of patients undergoing surgical intervention of their metastatic spine will still need a revision surgery. Revision surgery has been undertaken for surgical site infections and wound dehiscence, failure of instrumentation, and local recurrence/tumor progression [9]. In our study, 67% of the patient population undergoing revision surgery had tumor progression. The majority of patients undergoing a revision had renal cell carcinoma as the dominant primary malignancy type (41.7%), a cancer with an intermediate prognosis, which is also relatively radioresistant [15, 16].

Survival analysis revealed median survival in the revision group at 3.0 years, while the nonrevision group had 1.5 years. This approached statistical significance (*p* = 0.105). Metastasis to the spine carries a poor prognosis with time to death after surgery in most case series ranging from 11.3 to 15.4 months [17, 18]. Patients with better natural history of their primary pathology can be expected to have a longer survival time. This allows for an inherent bias, where there is increased lead-time for tumor progression or instrumentation failure prior to the patient's death. However, if the natural prognosis is not favorable, despite tumor progression or hardware failure, these patients might not undergo revision surgery secondary to their overall poor prognosis, poor performance status (ECOG), or generally poor medical condition.

In exploring prognostic factors, smoking was found to have a notable effect on the odds of needing a revision surgery. After controlling for confounding variables, including the use of chemotherapy, smoking increased the odds of needing a revision by 3.52 times compared to a nonsmoker. Smoking has been found to have a negative impact in many disease pathologies: both oncological [19, 20] and nononcological entities, including decreasing rate of union [21]. The latter is especially important in the case of spinal fusions, which are frequently used as a treatment modality for stabilizing the affected spinal segment. Smoking can delay union and allow for the development of hardware failure, necessitating a revision. Additionally, smoking has also been implicated in wound healing complications that can also necessitate a revision [22].

Thus, patients undergoing primary surgical treatment for their spinal metastatic disease should also be counseled on the inherent dangers of smoking. Not only does smoking result in the production of carcinogens that can further increase the aggressiveness and spread of the primary tumor [23, 24] but also the odds of the patient undergoing a revision for their spinal metastasis also substantially increase.

The finding of a lower incidence of failure and decreased need for revision in patients receiving chemotherapy on univariate analysis is similarly relatable to the concept of natural history. Patients with systemic options for therapy likely have a more significant disease burden and may have a shorter life expectancy; this could potentially create a lead-time bias in which the group not receiving chemotherapy could have a higher likelihood to undergo a revision.

The natural history of patients with metastatic spinal tumors appears to be grim with survival only for a few years after diagnosis at best. Patients undergoing revision surgery tend to have comparable survival times to those not undergoing a revision. Thus, if a patient demonstrates indications for undergoing a revision surgery, that is, develops a hardware failure, and if their overall medical condition allows them to undergo surgical intervention, they should be presented the option of undergoing a revision. Additionally, reoperation for recurrent metastatic tumors in patients with high-grade epidural spinal compression does not necessarily result in poor functional outcomes [25]. While smoking increases the odds of patients undergoing a revision procedure, a revision procedure does not necessarily decrease survival as compared to patients who only undergo a primary procedure.

For certain analyses, statistical significance was set at a *p* value of 0.10, due to the smaller sample size in certain categories. Additionally, due to the low prevalence of patients with this disease burden and the even lower prevalence of patients undergoing revision surgery, the patient numbers are, as expected, low and the study will be underpowered. Nonetheless, the relationships and conclusions postulated remain highly plausible. Additional studies with collaborations from multiple centers across vast geographical areas are needed to further confirm these findings. The biggest uncontrolled variable in studies such as this is the inconsistency of surgical indications and this may limit direct comparison of large groups of patients from multiple surgeons.

This study demonstrates some important differences when contrasted with a similar study by Quraishi et al. [14]. While the overall revision rate in this study was 2-fold higher, the mean survival time in the nonrevision group was two times longer and in the revision group it was nearly 4 times longer. This is unlikely to be a treatment effect but rather an observation that may be related to tumor-specific factors, surgical selection, or other indeterminate factors. Another major difference in this group is that 20 of 31 operations in the study by Quraishi [14] were performed in the same hospitalization (early reoperation) versus our study, where there were no early reoperations (mean of 685 days with a range of 102–1666 days).

## 5. Conclusion

This study primarily compares the survival outcomes of patients undergoing revision surgery versus those undergoing only a primary procedure and explores characteristics that might influence patients needing a revision at a single institution. This particular study design may allow for more homogeneity of indications and technique. This will provide additional predictive information for the surgeon to counsel the patients and pursue revision surgery when indicated.

## Figures and Tables

**Table 1:** Patient and tumor characteristics.

	***Revision (n *= 12)**		***No revision (n* = 43)**		***pvalue***
	***n***	***%***	***n***	***%***	
**Gender**					*>.999* ^*a*^
Male	7	58.3	26	60.5	
Female	5	41.7	17	39.5	
**Race**					*>.999* ^*a*^
Caucasian	9	75.0	31	72.1	
African American	2	16.7	7	16.3	
Asian	1	8.3	3	7.0	
Other	0	0.0	2	4.7	
**Smoker**					*.055* ^*a*^
Yes	9	75.0	18	41.9	
No	3	25.0	25	58.1	
**Primary malignancy type**					*.323* ^*a*^
Renal cell carcinoma	5	41.7	10	23.3	
Melanoma	1	8.3	4	9.3	
Colorectal	0	0.0	6	14.0	
Breast	2	16.7	2	4.7	
Multiple myeloma	0	0.0	7	16.3	
Lung	1	8.3	3	7.0	
Other	3	25.0	11	25.6	
**Vertebral levels**					
C-Mets	3	25.0	8	18.6	*.689* ^*a*^
T-Mets	9	75.0	30	69.8	*>.999* ^*a*^
L-Mets	1	8.3	12	27.9	*.255* ^*a*^
**Pain**					*.566* ^*a*^
Yes	12	100.0	39	90.7	
No	0	0	4	9.3	
**Spine Mets at initial presentation**					*.050* ^*a*^
Yes	0	0.0	12	27.9	
No	12	100	31	72.1	
**ASIA motor score at first visit**					*.826* ^*a*^
E	11	91.7	34	79.1	
D	1	8.3	5	11.6	
C	0	0.0	4	9.3	
**Allograft**					*.147* ^*a*^
Yes	11	91.7	29	67.4	
No	1	8.3	14	32.6	
**Chemo**					*.064* ^*a*^
Yes	9	75.0	41	95.4	
No	3	25.0	2	4.6	
**Radiation**					*.255* ^*a*^
Yes	11	91.7	31	72.1	
No	1	8.3	12	27.9	
**Preop. embo.**					*.730* ^*a*^
Yes	4	33.3	12	27.9	
No	8	66.7	31	72.1	
**Extraspinal Mets**					*.429* ^*a*^
Yes	8	66.7	35	81.4	
No	4	33.3	8	18.6	
**Age (years)**					*.741* ^*b*^
Mean (SD)	59.1 (7.5)		58.1 (13.3)		
**Spine burden**					*.546* ^*c*^
Median (IQR)	1.0 (1.0-3.5)		1.0 (1.0-2.0)		

**Table 2:** Reasons for revision.

	***Revision***	
**Revision reason**	***n***	***%***
Tumor progression	8	66.7
Nonunion	2	16.66
Wound dehiscence	1	8.33
Construct failure	1	8.33

**Table 3:** Univariate regression analysis for undergoing revision surgery.

**Variable**	**Odds ratio**	***p* value**	**95% confidence interval**	
Age	1.007	.803	0.954	1.063
Race		>.999		
* Caucasian versus other*	*>999.999*	.985	*<0.001*	*>999.999*
* African American versus other*	*>999.999*	.985	*<0.001*	*>999.999*
* Asian versus other*	*>999.999*	.984	*<0.001*	*>999.999*
Sex				
* Male versus female*	*0.915*	*.894*	*0.249*	*3.360*
Smoker				
* Smoker versus nonsmoker*	*4.166*	*.052*	*0.987*	*17.589*
Primary malignancy type		.956		
* RCC versus other*	*1.833*	*.904*	*0.346*	*9.719*
* Melanoma versus other*	*0.917*	*.924*	*0.073*	*11.577*
* Colorectal versus other*	*<0.001*	*.949*	*<0.001*	*>999.999*
* Breast versus other*	*3.667*	*.884*	*0.354*	*38.029*
* Multiple myeloma versus other*	*<0.001*	*.946*	*<0.001*	*>999.999*
* Lung versus other*	*1.222*	*.916*	*0.091*	*16.429*
Preop. ambulatory status				
* IND/AMB assist versus wheelchair/bed rest*	*>999.999*	*.957*	*<0.001*	*>999.999*
Radiation				
* Yes versus no*	*4.258*	*.187*	*0.495*	*36.659*
Chemo				
* Yes versus no*	*0.146*	*.051*	*0.021*	*1.008*
Extraspinal Mets				
* Presence versus absence*	*0.457*	*.282*	*0.110*	*1.901*

**Table 4:** Multivariable regression analysis for undergoing revision surgery.

**Variable**	**Odds ratio**	***p* value**	**95% confidence interval**	
Smoker				
* Smoker versus nonsmoker*	*3.518*	*.096*	*0.801*	*15.457*
Adjuvant chemotherapy				
* Yes versus no*	*0.196*	*.110*	*0.027*	*1.450*
